# The Social Legitimacy of Pain: Protocol for a Systematic Literature Review

**DOI:** 10.3390/healthcare10122400

**Published:** 2022-11-29

**Authors:** Gema Serrano-Gemes, Rocío Vizcaíno-Cuenca, Rafael Serrano-del-Rosal

**Affiliations:** 1 Departamento de Enfermería, Facultad de Ciencias de la Salud de Ceuta, Universidad de Granada (UGR), 51001 Ceuta, Spain; 2 Instituto de Estudios Sociales Avanzados (IESA), Consejo Superior de Investigaciones Científicas (CSIC), 14004 Córdoba, Spain; 3 Departamento de Metodología de las Ciencias del Comportamiento, Facultad de Psicología, Universidad de Granada (UGR), 18071 Granada, Spain

**Keywords:** pain, social legitimacy, systematic review, protocol

## Abstract

*Introduction*: Pain is a perception conditioned both by the subjective experience of the sufferer and their social context. A preliminary literature search suggests that, today, information about the social legitimacy of pain is scarce, although it is known that this phenomenon is an element that is closely linked to the type of pain and contributes to the sufferer’s experience. Thus, our objective is to explore how the social legitimacy of pain is tackled in the scientific literature. *Methods*: This study is a protocol for a systematic literature review where six databases were reviewed: Pubmed, Web of Science, Scielo, Scopus, PsycInfo, and CINAHL, were searched for papers dealing with the social legitimacy of pain from any discipline/study design. The obtained papers will be exported to Zotero, where the duplicates will be deleted. Later, the studies of interest will be selected, first on the basis of their titles/abstracts, and, later, on the complete text. This process will be carried out in pairs. Finally, the data of interest will be extracted, analyzing their quality, to finally make a qualitative analysis of the results. *Discussion*: This will be the first review to systematically explore the available evidence on the social legitimacy of pain. Therefore, it will be able, not only to extend the theoretical knowledge on this phenomenon, but also to extend its visibility, which will make it possible for the social legitimacy of pain to be studied from different disciplines and fields, thus improving the way it is approached.

## 1. Introduction

Pain is an unpleasant experience which, in one way or another, every person experiences throughout their life, causing serious effects at least at the clinical, economic, and social levels. A recent survey about the social perception of pain in Spain showed that almost 70% of the population has suffered chronic pain at some point in their lives, thus affecting their wellbeing and quality of life at the same time [1]. In this regard, Henschke et al. [2] brought to light that the experience of pain interferes negatively in a sufferer’s everyday life, such as in their general perception of health, and their interactions with other people. In fact, previous studies have highlighted the positive association between the pain experienced with loneliness, isolation, and social dissatisfaction [3,4], for instance. In another study carried out in Europe, about the consequences of pain, more than half of the people surveyed revealed their difficulties to get to sleep, even pointing out that almost one fourth had quit their jobs [5]. All these data reveals the importance and need to conduct research into the different dimensions of pain. However, although many studies have intended to move forward in the understanding and treatment of pain from a biomedical perspective [6,7], it was not until the last decade that research, proposing and demanding an approach to pain from a multidimensional perspective, has emerged [8,9].

This is why, in order to contextualize the relevance of this research topic, it bears providing a brief account of the theoretical precedent studies on pain. Up until the mid-20th century, pain had only been studied from a physiological point of view [10]. It was after the gate control theory of pain that the exclusive understanding of pain as a primary sensation was abandoned, and the influence of the experience of learning about pain began to be considered [11]. From then on, pain has been conceived as a perceptive experience with three dimensions: the sensory (biological elements, which partially determine the location, duration, and intensity of pain), motivational–affective (which involves how people experience and deal with pain), and cognitive dimensions (which include cognitions, such as cultural values, appraisal, and hypnotic suggestion) [12]. In this paper, our conceptual basis is the definition proposed by the International Association for the Study of Pain (IASP), which is widely accepted in academia and defines pain as: “an unpleasant sensory and emotional experience associated with, or resembling that associated with, actual or potential tissue damage” [13] (p. 1977). This conception points out the subjectivity of the painful experience and underlines the acquisition of knowledge on pain through experiences in a social context [13], without denying the preceding nature of harmful stimulation, even if it is not discernible. In addition, it is assumed that pain has an intersubjective nature, since it is socially learned and shared [14]. Consequently, due to the complexity of its notion, the study of pain must be founded on a biopsychosocial model, which considers the joint influence of biological, psychological, and social factors [15].

Regarding this wide view on the study of pain, some previous research focused on variables, such as location, intensity, duration, etiology, or the impact on people’s social life [9,16]. However, research on the social perception of pain has been scarce, despite the fact that pain, apart from being an unquestionable physical and anatomical element [11], also has an intersubjective nature that leads to a different perception and interpretation on the basis of cultural and social factors [15]. In this sense, the way in which people perceive pain socially has an influence insofar as they empathize and support the sufferer, thus legitimizing the pain they suffer in accordance with a socially constructed hierarchy based on elements, such as the pain’s nature, type, and/or intensity, or its “meaning” (e.g., the brain activates the same areas when there is a physical or an emotional pain, but on a social level they are perceived as different due to social, historical, and cultural aspects) [14]. Likewise, certain types of pain are stigmatized as a result of believing that the individuals are responsible for suffering them [17] or because of the sufferer’s social characteristics [1]. Along these lines, although the neuronal mechanisms are not different according to the different types of pain [18], differences may be observed in their social perception and recognition.

For all these reasons, it is essential to achieve a complete knowledge that includes the physiological, psychological, and sociological elements of pain, so that it may be better understood, correctly interpreted, and thus, its consequences may be palliated. However, the attention that has been paid by research to the social legitimacy of pain is unknown, and that is why our research question is: how is the social legitimacy of pain tackled in the scientific literature?, focusing on two aspects of interest, according to the PICO framework: the population “P” and the outcome of interest “O” [19]. Therefore, the “P” will refer to all the types of scientific studies/approaches on the topic, while the “O” will focus on the social legitimacy of pain. So, our main objective will be to explore how the legitimacy of pain is tackled in the scientific literature, identifying the types of study, the scientific and geographic fields where this concept was dealt with, analyzing the contexts of health and/or illness where it was used, and describing the sociodemographic groups where it was studied.

## 2. Methods

### 2.1. Design

The protocol for a systematic literature review, which was developed considering PRISMA-P. [19,20] (Supplementary Material S1). This protocol was registered in PROSPERO 2021 (CRD42021251018).

### 2.2. Exploratory Preliminary Study

**Figure 1:**
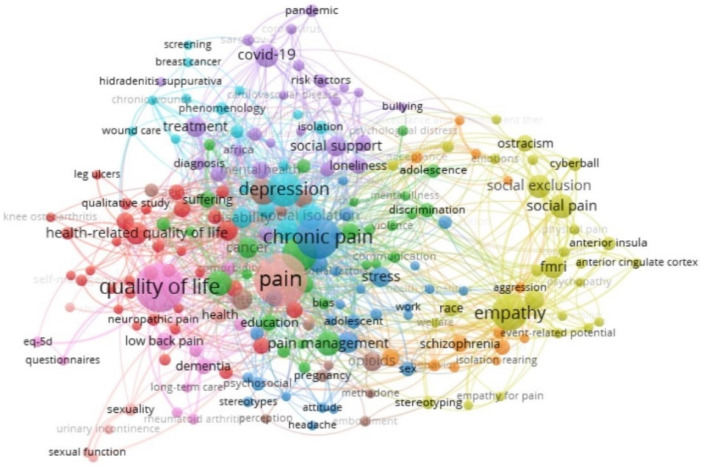
Chart showing the main nodes (keywords) applying the original unfiltered strategy on Scopus (Using the VOSviewer software (Centre for Science and Technology Studies, Leiden University, The Netherlands. Version 1.6.16.)).

**Figure 2:**
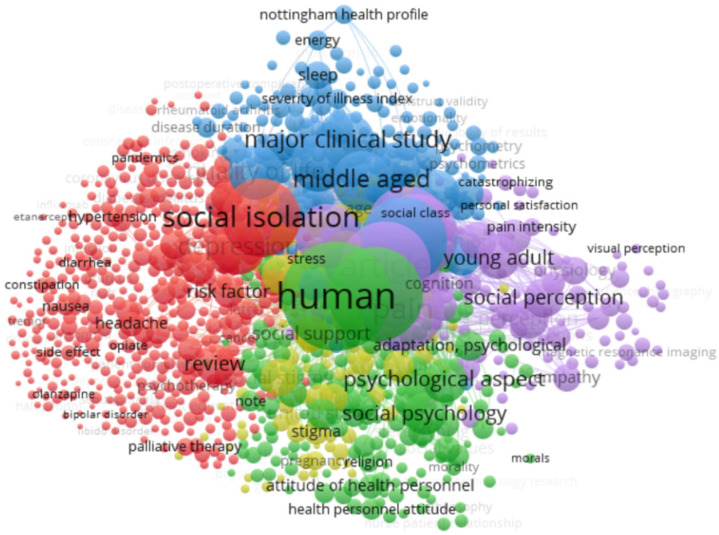
Chart showing the main nodes (keywords) applying the final filtered search strategy on Scopus (Using the VOSviewer software (Centre for Science and Technology Studies, Leiden University, The Netherlands. Version 1.6.16.)).

With the aim of studying the appropriateness of carrying out a systematic review of this topic, a preliminary study of the literature was made first. This process took place in two stages: first, in order to avoid the unnecessary duplication of systematic reviews, following the recommendations by Moher [21], we checked that there were no previous works tackling our topic of interest or research objectives. To this end, a small preliminary study was made on three specific databases of review protocols/systematic reviews (Cochrane Library, Joanna Briggs Institute Evidence Synthesis, and the PROSPERO International prospective register of systematic reviews), which were accessed using the keywords “legitimacy” and “pain”, and no study linked to our research question was found. Secondly, a small analysis was conducted in order to find the best possible search strategy. To do this, a first search strategy was designed, which was piloted in: PubMed, Scopus, and the Web of Science core collection. Later, the records of each search were exported and analyzed using the VOSviewer software (Centre for Science and Technology Studies, Leiden University, The Netherlands. Version 1.6.16.) (https://www.vosviewer.com/ accessed on 17 May 2021). This is a program developed primarily to build and visualize bibliometric networks. However, VOSviewer is also capable of performing a text mining process, which allows to create and visualize co-occurrence networks of relevant terms extracted from the literature. The latter functionality, the study of keyword co-occurrence, is the one used in this review protocol. Thus, this analysis made it possible to check the existence of any thematic confusion through the visualization of the keywords in each search and the relationships between these terms. Thanks to this, it was possible to adjust the search strategy, eliminating some of the terms used at first because of the thematic confusion they created or their apparent poor relationship with the social legitimacy of pain. Finally, the new search strategy was tested on the three piloted databases, then dumping the results again in VOSviewer, and checking the improvement of the new search strategy, which was later adjusted to all the databases that will be used. As an example, Figure 1 shows the map generated with the data from Scopus using the original strategy, while Figure 2 shows the map obtained using the final refined strategy for the same database.

Source: VOSviewer software (Centre for Science and Technology Studies, Leiden University, The Netherlands. Version 1.6.16.) (https://www.vosviewer.com/ accessed on 17 May 2021). Strategy prepared on Scopus (Designed by the authors).

### 2.3. Information Sources

The following databases were consulted for this study: PubMed, Web of Science Core Collection (through Web of Science, managed by the Spanish Foundation for Science and Technology), Scielo Citation Index (through Web of Science, managed by the Spanish Foundation for Science and Technology), Scopus, APA PsycInfo (through ProQUEST), and CINAHL Complete (through EBSCOhost), searching them from their beginning until the present (date on which the searches were carried out: 1 March 2022).

### 2.4. Search Strategies

The designed search strategies are based on two concepts of interest: “pain” and “social legitimacy”. The first one was used whenever possible, using the specific descriptor of the relevant database. For the second concept, as no specific/concrete descriptor defining it was found, we decided to use different terms linked to the concept of legitimacy, which are tackled in our study, using specific descriptors from the different databases and other undefined terms. The different search strategies used may be consulted in Table 1.

### 2.5. Inclusion and Exclusion Criteria

In this review, pain will be understood as a situated experience, both in the person and in its context. The manifestations or causes of pain may be physical or emotional, psychological, social, etc. Thus, pain will be looked at from a holistic point of view. As for the social legitimacy of pain, it will not be understood as the legitimacy of pain itself, but also as its respect, credibility, recognition, comprehension, will, and visibility, also those studies dealing with the legitimacy of pain in a negative way, i.e., the lack of legitimacy of pain will be considered. In addition, it will be understood that legitimacy lies in different aspects of the experience of pain: in the person itself, in society, in the healthcare, and/or social systems, etc. With this approach, the legitimacy/illegitimacy is studied: (1) in a direct way, for example through how people experience or feel legitimacy/illegitimacy; (2) indirectly, for example through the descriptions made by external observers. A synthesis of the inclusion and exclusion criteria to be used is shown in Table 2.

### 2.6. Study Selection

In order to improve our reviews reproducibility, quality, and transparency, [22] it is important to point out that the studies retrieved from the included databases will be exported to the Zotero bibliographic management software, in order to manage and purge them and eliminating duplicates. Later, the studies will be reviewed, according to their titles and abstracts, or just their titles, if there is no abstract, so as to verify that they comply with the inclusion criteria. In the cases of books or theses, their tables of contents will also be consulted in order to tackle any specific chapter(s) of interest. Those that do not comply with the criteria will be excluded, while those complying with them will be reviewed in their entirety to make sure that they still comply with the criteria. Both stages, the one that involves checking the title/abstract, and the one where the entire text is checked, will be carried out independently by two researchers, who will later discuss and reach an agreed decision in the cases in which they do not agree. This complete dual review method was chosen because evidence shows, that by using it, the accuracy of the study selection in the systematic reviews is improved [23]. This study selection process will be shown in a flow chart, following the updated model from PRISMA 2020 [24].

### 2.7. Data Extraction

In order to extract information, following the recommendations from the Guide to Writing a Qualitative Systematic Review Protocol by Butler et al. [25], a specific tool for data extraction is created, adapted to the objectives set in this review. Our tool will include information about the authors, year, country, research field, objectives, study design and methodology, population, health context and/or studied illness, a short summary of the results/conclusion, information about where it was published, and a check in whether the document was peer-reviewed. The data extraction process will be conducted by two reviewers independently, who will discuss and reach an agreed decision in the cases in which they do not agree. Additionally, independently, the same two reviewers will check the usefulness of the tool, by conducting a pilot study of 2–5 studies.

### 2.8. Quality Assessment

The creators of the PRISMA 2020 tool highlights the importance of assessing the risk of bias, which is different from the quality assessment [26]. These authors explained that the literature points out that quality does not only include the study of the elements, which may bias the findings, but also the applicability, imprecision, completeness, or ethics [26]. Taking this into account, this review protocol was chosen to assess the quality and not the risk of bias, because the study of the abovementioned aspects is vital to provide a much more complete response to our review objective, whose motive is descriptive, synthetic, and intends to be an exhaustive exploration of the literature.

As for the specific assessment method to be used, it bears mentioning that this review expects to obtain a great variety of studies, with great heterogeneity in design and methodology. This circumstance forces us to make two proposals for a quality assessment. As a first option, as long as there is not much variability in the study designs, quality will be assessed through the appropriate tools for such designs proposed by EQUATOR (https://www.equator-network.org/ accessed on 17 May 2021). On the contrary, if there is a great heterogeneity in the methodology and the designs found, and no appropriate tools are found in EQUATOR to assess them, quality will be assessed through our own tool, created for this purpose, since the methodological heterogeneity will make it difficult to compare the different studies due to the different tools used or even the lack of tools to perform this assessment. This tool will include information about the methodological aspects (record, design report, methodology, population, sampling, result or object of interest variables), ethical aspects (informed consent and ethics committees), and a declaration of sensitive information (conflicts of interest and funding). The answers for each section are YES, NO, INCOMPLETE, NOT SHOWN, NOT APPLICABLE, having to justify in writing any question different from YES. This approach is somewhat inspired by the Cochrane Handbook for Systematic Reviews of Interventions [27]. This tool will be independently piloted by two reviewers, using 2–5 studies to this end.

Finally, either if the first or the second proposal are used, the quality of all the studies included in our review will be assessed. The quality assessment process will be performed by two reviewers independently, who will discuss and reach an agreed decision in the cases in which they do not agree. The information on the quality of the studies will be included in a summary table.

### 2.9. Data Synthesis

Due to the great variability of the types of studies that are expected to be obtained, which will probably present a great heterogeneity of methodologies, the synthesis of the obtained information will be made through a descriptive analysis of the studies. All the information of interest will preferably be shown in tables, displaying the relevant information in a simple and clear manner.

## 3. Discussion

This is the first systematic review that addresses the social legitimacy of pain. This is undoubtedly a great opportunity. As we strongly believe that understanding how pain is legitimized/illegitimized at the societal level, it will allow it to be better treated and addressed at a professional, research and, ultimately, society level. At a professional level, this will allow a real biopsychosocial approach to pain. This will be possible because it will not only allow for an understanding into the social implications that pain can trigger in individuals and their environment, but also how the social environment causes and/or modulates the perception of pain and defines what can be considered as such. On the other hand, in terms of research, this study attempts to put the importance of the study of the legitimacy of pain on an international agenda. As this is one of the key elements of a scarcely studied social dimension of pain. Finally, at a societal level, this study will help to know which and what are the most important sources and barriers to the social legitimacy of pain. Since this is the first step to be able to implement studies and policies, which tries to alleviate the psychological and social impairment of citizens who, in addition to feeling pain, are ignored and not believed, elevating their pain to the category of serious human suffering. Therefore, we hope that our future review will lay the groundwork for this new line of research, which we believe is not only novel, but also extremely necessary.

Thus, thanks to this systematic review it will be possible to achieve a deep understanding of how the social legitimacy of pain is tackled today. In this way, this review will lay a solid foundation into any studies dealing with this phenomenon, which, despite being complex and being hardly studied so far, as already mentioned, has a direct effect on the sufferer’s experience and threshold [1]. Therefore, we believe that this review will provide a necessary visibility to the social legitimacy of pain, which will make it possible to go deeper into its study via different disciplines and fields, including applied studies, which in the future, will enable the designing of more efficient treatments, as well as healthcare services, in a more comprehensive way. In addition, in our particular case, this review will also make it possible to continue with this line of research of our team, which has extensive experience on the matter [1,14,28], apart from presently having an open funded project where this topic is studied and developed in depth. Furthermore, it bears pointing out that this review protocol also informs, in detail, all the decisions dealing with aspects, tools, and the methodology, which will be taken throughout our review. To this end, PRISMA-P was taken into account, with the aim of improving the reliability and methodological quality of our future review [20].

Finally, it is necessary to mention two limitations that our future review has: a language limitation (only documents written in English and Spanish will be taken into account) and a (foreseeable) limitation of access to documents (due to the fact that there may be documents, which are impossible for us to have access to, with our means/possibilities). Although we recognize the possible limitations that these aspects may cause in our review, the authors do not believe that their influence on the results will be significant. Moreover, as the literature will be reviewed, not only in the most prevalent language in science, such as English, but another language, Spanish, was also added. Furthermore, with regards to the access to the literature, all available means will be used through the research team that is part of the research project in which this systematic review is framed.

## Figures and Tables

**Table 1:** Search strategies according to each database.

Database	Strategies	Filters	Results
Pubmed	(((pain[MeSH Terms])) AND ((((((((((“Cultural Values”) OR ((Social Perception[MeSH Terms]))) OR ((Social Stigma[MeSH Terms]))) OR ((Social Values[MeSH Terms]))) OR ((Social Conformity[MeSH Terms]))) OR ((Social Norms[MeSH Terms]))) OR ((Stereotyping[MeSH Terms]))) OR ((Prejudice[MeSH Terms]))) OR ((Social Discrimination[MeSH Terms]))) OR ((Social Isolation[MeSH Terms])))) NOT ((((Analgesics [MeSH Terms])) OR ((Diagnostic Imaging [MeSH Terms]))) OR ((Euthanasia [MeSH Terms])))	Spanish, English, Human	776
Cinahl Complete	(MH “Pain+”) AND ((MH “Cultural Values”) OR (“Social Perception”) OR (MH “Stigma”) OR (MH “Social Values+”) OR (MH “Social Conformity”) OR (MH “Social Norms”) OR (MH “Stereotyping”) OR (MH “Prejudice+”) OR (MH “Discrimination+”) OR (MH “Social Isolation+”)) NOT ((MH “Euthanasia+”) OR (MH “Analgesics+”) OR (MH “Diagnostic imaging+”) OR (MH “Animals+”))	Spanish, English	905
APA PsycInfo	MAINSUBJECT.EXACT.EXPLODE(“Pain”) AND (MAINSUBJECT.EXACT(“Cross Cultural Differences”) OR MAINSUBJECT.EXACT.EXPLODE(“Social Perception”) OR MAINSUBJECT.EXACT(“Social Values”) OR (“Social Conformity”) OR MAINSUBJECT.EXACT(“Social Norms”) OR MAINSUBJECT.EXACT.EXPLODE(“Social Acceptance”) OR MAINSUBJECT.EXACT(“Stereotyped Attitudes”) OR MAINSUBJECT.EXACT.EXPLODE(“Prejudice”) OR MAINSUBJECT.EXACT.EXPLODE(“Social Discrimination”) OR MAINSUBJECT.EXACT.EXPLODE(“Social Isolation”)) NOT (MAINSUBJECT.EXACT.EXPLODE(“Animals”) OR MAINSUBJECT.EXACT.EXPLODE(“Narcotic drugs”) OR MAINSUBJECT.EXACT.EXPLODE(“Analgesic Drugs”) OR MAINSUBJECT.EXACT(“Euthanasia”) OR MAINSUBJECT.EXACT.EXPLODE(“Neuroimaging”))	Spanish, English, Human	571
Scopus	(INDEXTERMS(“pain”)) AND ((INDEXTERMS(“Cultural Values”)) OR (INDEXTERMS(“Social Perception”)) OR (INDEXTERMS(“Social Stigma”)) OR (INDEXTERMS(“Social Values”)) OR (INDEXTERMS(“Social Conformity”)) OR (INDEXTERMS(“Social Norms”)) OR (INDEXTERMS(“Stereotyping”)) OR (INDEXTERMS(“Prejudice”)) OR (INDEXTERMS(“Social Discrimination”)) OR (INDEXTERMS(“Social Isolation”))) AND NOT ((TITLE-ABS-KEY(“euthanasia”)) OR (TITLE-ABS-KEY(“analgesic*”)) OR (TITLE-ABS-KEY(“opioid*”)) OR (TITLE-ABS-KEY(“Diagnostic Imaging”)) OR (TITLE-ABS-KEY(“animal*”)))	Spanish, English	1568
Web of Science	TS = (“pain”) AND (TS = (“Cultural Values”) OR TS = (“Social Perception”) OR TS = (“Social Stigma”) OR TS = (“Social Values”) OR TS = (“Social Conformity”) OR TS = (“Social Norms”) OR TS = (“Stereotyping”) OR TS = (“Prejudice”) OR TS = (“Social Discrimination”) OR TS = (“Social Isolation”)) NOT (TS = (“Euthanasia”) OR TS = (“Analgesic * ”) OR TS = (“Opioid * ”) OR TS = (“Animal * ”) OR TS = (“Diagnostic Imaging”))	Spanish, English	933
SciELO	TS = (“pain”) AND (TS = (“Cultural Values”) OR TS = (“Social Perception”) OR TS = (“Social Stigma”) OR TS = (“Social Values”) OR TS = (“Social Conformity”) OR TS = (“Social Norms”) OR TS = (“Stereotyping”) OR TS = (“Prejudice”) OR TS = (“Social Discrimination”) OR TS = (“Social Isolation”)) NOT (TS = (“Euthanasia”) OR TS = (“Analgesic * ”) OR TS = (“Opioid * ”) OR TS = (“Animal * ”) OR TS = (“Diagnostic Imaging”))	Spanish, English	33

Source: Prepared by the authors. Date on which the searches were carried out: 1 March 2022.

**Table 2:** Inclusion and exclusion criteria *.

Inclusion Criteria		
(a) Studies dealing with the social legitimacy of pain from any discipline or approach. (b) Studies written in English or Spanish. (c) Studies conducted in humans.		
Exclusion Criteria		
Legitimacy	Pain	Methodological criteria
(a) Studies on the legitimacy of euthanasia or the legitimacy of using certain drugs/medicines (for example: opioids). (b) Studies on the legitimacy or social legitimacy of any other process, concept, or element different from pain (in any of its dimensions).	(c) Studies focusing on pain (in any of its dimensions) without considering its social legitimacy. (d) Studies focusing on painful processes with marked suffering, but understood as a process in themselves, for instance, mourning. (These studies will be excluded if they do not specifically focus on the social legitimacy of pain). (e) Studies linked to the efficiency of drugs/medicines to relieve pain. (f) Studies linked to the efficiency of diagnostic tests.	(g) Studies conducted in animals. (h) Studies whose complete text cannot be accessed.

* When we refer to “studies” we are referring to all types of scientific documents, without distinction by design or methodology. Sources: prepared by the authors.

## Data Availability

Not applicable.

## Supplementary Materials

The following supporting information can be downloaded at: www.mdpi.com/article/10.3390/healthcare10122400/s1, Supplementary Material S1.
